# Dynamic causal modeling of spontaneous fluctuations in skin conductance

**DOI:** 10.1111/j.1469-8986.2010.01052.x

**Published:** 2011-02

**Authors:** Dominik R Bach, Jean Daunizeau, Nadine Kuelzow, Karl J Friston, Raymond J Dolan

**Affiliations:** a Wellcome Trust Centre for Neuroimaging, University College LondonLondon, United Kingdom; b Institute for Psychology and Ergonomics, Technical University of BerlinBerlin, Germany

**Keywords:** SCR, Galvanic skin responses, GSR, Electrodermal activity, EDA

## Abstract

Spontaneous fluctuations (SF) in skin conductance are often used to index sympathetic arousal and emotional states. SF are caused by sudomotor nerve activity (SNA), which is a direct indicator of sympathetic arousal. Here, we describe a dynamic causal model (DCM) of how SNA causes SF, and apply variational Bayesian model inversion to infer SNA, given empirically observed SF. The estimated SNA bears a relationship to the number of SF as derived from conventional (semi-visual) analysis. Crucially, we show that, during public speaking induced anxiety, the estimated number of SNA bursts is a better predictor of the (known) psychological state than the number of SF. We suggest dynamic causal modeling of SF potentially allows a more precise and informed inference about arousal than purely descriptive methods.

Changes in skin conductance are common indicators of sympathetic arousal whose proximal cause is changing activity of sweat glands innervated by the sympathetic branch of the autonomic nervous system (ANS). The number of spontaneous fluctuations (SF) in skin conductance is among the most widely used measures of tonic ANS activity (for an overview, see Boucsein, 1992) and is thought to reflect variations in arousal stemming from a variety of cognitive and emotional processes. SF are sensitive to small changes in arousal (Boucsein, 1992), and play an important role in inferring stress (Boucsein, 1992) and anxiety (Erdmann & Baumann, 1996).

SF occur in the absence of external events, and are preceded by firing bursts of sudomotor nerve activity (SNA), innervating the respective skin region (Macefield & Wallin, 1996; Nishiyama, Sugenoya, Matsumoto, Iwase, & Mano, 2001; Ogawa & Sugenoya, 1993;). On this basis, a facility to directly assess SNA instead of SF should provide a closer approximation to underlying autonomic states. In the absence of invasive methods this can, in principle, be realized using model inversion methods that map observed fluctuations in skin conductance to underlying SNA. This approach is now frequently employed in neuroimaging within the framework of dynamic causal modeling (DCM) (Friston, Harrison, & Penny, 2003). At the heart of DCM is a causal model, also referred to as a generative or forward model, which describes a mapping from underlying causes (i.e., neural states) to empirical observations (e.g., BOLD response, EEG waveform, or SF).

In the case under consideration here, the model predicts observed SF, given SNA. Inverting the causal model yields the reverse mapping from observations to the (most likely) underlying causes; in our case, the inversion SF ↦ SNA describes the (most likely) generative sudomotor nerve activity, given observed skin conductance. The key difference between previously proposed models for event-related skin conductance changes where event timing is known (Bach, Flandin, Friston, & Dolan, 2009; Lim et al., 1997;) and the model considered here is that both timing and amplitude of SNA bursts have to be estimated from the data. Deconvolution methods afford such estimates, as they try to recover the precise SNA time series from the skin conductance data (Alexander et al., 2005; Benedek & Kaernbach, 2009;). Our approach represents an informed Bayesian deconvolution, which rests on parameterizing the SNA in a way that allows a quantitative description of the underlying state. This parameterization places constraints on inferred SNA and decreases the degrees of freedom of the model, which increases the precision of model parameter estimates, especially when analyzing noisy data.

In this paper, we describe a DCM for SF, with two goals. First, we wanted to show that a DCM for skin conductance can explain data from different individuals and experiments and to motivate further research into the underlying physiology. Second, we sought to establish external validity of the model. We hypothesized that estimates of the underlying autonomic state based on DCM predict (known) psychological states more accurately than estimates from conventional methods. To allow other researchers to perform similar evaluations, the method is included as function scr_sf_dcm.m in the software suite SCRalyze, which is freely available under the GNU general public license from http://scralyze.sourceforge.net.

## Methods

### Forward Neural Model

**Figure 1:**
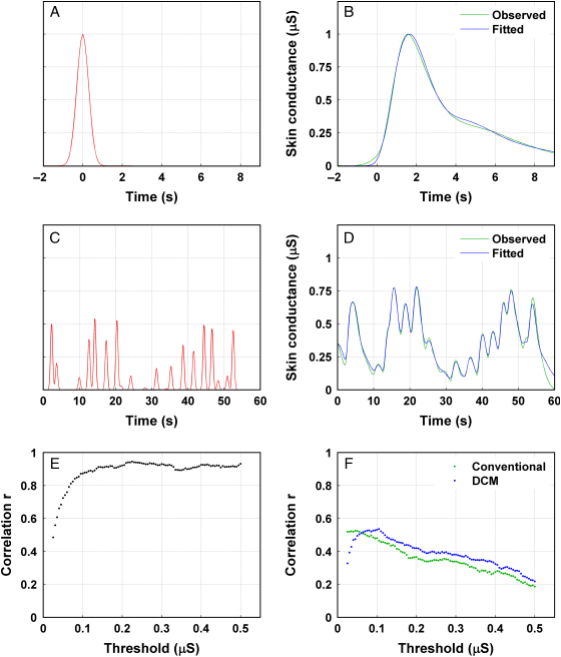
(A) Modeled sudomotor nerve firing burst of unit amplitude that is assumed to cause a spontaneous fluctuation of 1 μS amplitude. (B) Green: canonical response function for a single spontaneous fluctuation, derived from the first dataset by using an uninformed finite impulse response model and specifying SF onsets using conventional (semi-visual) analysis. Blue: analytical approximation to this function obtained by optimizing the parameters of a third-order ordinary differential equation using a Bayesian inversion scheme. (C) Estimated SNA for a sample epoch. (D) Empirical skin conductance for this epoch, and estimated skin conductance obtained by DCM using the estimated SNA shown in panel C and the SF function shown in panel B. (E) Correlation between the number of responses revealed by conventional analysis and DCM as a function of the threshold for detecting a response. (F) External validity for the number of responses revealed by conventional analysis and DCM inversion as a function of the threshold for detecting a response.

The duration and shape of SNA firing bursts is not well described; one study reported a duration of 637±37 ms (Macefield & Wallin, 1996), although from figures in this and other reports (Nishiyama et al., 2001; Ogawa & Sugenoya, 1993;) it seems that burst duration can extend up to 1.5–2 s. The number of SNA bursts differs between these studies, from 3±0.5 per minute (Nishiyama et al., 2001) to 22±4 per minute (Macefield & Wallin, 1996). In the absence of precise knowledge about SNA bursts, we make the simplifying assumption that they differ in amplitude but have a fixed temporal profile, and modeled them as Gaussian bump functions with a standard deviation of 0.3 s and a maximum frequency of 30 bursts per minute. Figure 1A shows a burst with unit amplitude (i.e., that would cause an SF with amplitude of 1 μS).

### Forward Response Model

No simultaneous recordings of SF and SNA have addressed how the shape of the ensuing SF relates to bursting, but there is some indirect evidence that SF have a largely constant shape (Bach, Friston, & Dolan, 2010) and that overlapping skin conductance changes build up in a linear fashion (Bach, Flandin, Friston, & Dolan, 2010), such that SF can be regarded as a product of a linear time-invariant system, although this needs to be validated in physiological experiments. The former paper also describes an impulse response function or convolution kernel reflecting the canonical shape of an individual SF at a phenomenological level (i.e., not derived from a biophysical model, but from physiological observations). Note that this canonical SF function (shown in Figure 1B) has a slightly biphasic decay, in line with a recent model of event-related skin conductance responses (Bach, Flandin, Friston, & Dolan, 2010). This biphasic response is predicted by the qualitative pore valve model (Edelberg, 1993), where the steep rise and fall of the skin conductance are caused by rapid opening and closing of sweat duct pores, while a slower recovery is afforded by evaporation of remaining sweat on the skin.

Our DCM models the relationship between SNA and SF as a linear time-invariant convolution (Bach, Flandin, Friston, & Dolan, 2009). This was specified in terms of an impulse response function for SF developed previously (Bach, Friston, & Dolan, 2010) and modeled here with a third-order ordinary differential equation (ODE). Figure 1B shows the empirically derived canonical function and its analytic ODE approximation; see the Appendix for details. This ODE is formally equivalent to a biphasic exponentially decaying convolution kernel and therefore captures the biphasic effects described above.

The assumed (Gaussian) form of SNA firing bursts and the subsequent ODE convolution that generates observed SF constitute the DCM. The resulting generative model assumes that, in the absence of any SNA, the skin conductance returns to zero, which is not normally the case in SF recordings. We were not interested in this baseline, or its slow drifts, because they are determined not only by SNA but also by peripheral factors (Boucsein, 1992). To remove this confounding data feature, we apply our models to skin conductance time-series that are high-pass filtered during recording (where the lowest value of each segment is subtracted). Sustained SF are modeled by repeated, low-amplitude SNA bursts. This is biophysically plausible, but can lead to discrepancies between the estimated number of bursts and the SF number assessed by (semi-)visual methods.

### Datasets

We reanalyzed one previously published (Bach & Erdmann, 2007, 2008) and one unpublished (for a review, see Erdmann & Janke, 2008) dataset from the same laboratory, both of which are based upon a similar paradigm. Dataset 1 served as a training dataset, which we used to optimize the parameters of the ODE that determine the shape of the implicit convolution kernel (see Appendix), and the amplitude threshold for counting responses. Dataset 2 served as an independent validation dataset, which was analyzed using the parameters from the first dataset.

Dataset 1 contained four measurements from each of 40 healthy male university students (18–35 years) who participated in a public speaking anticipatory anxiety paradigm with a repeated-measures factorial design. The main focus of this experiment was the interaction of habitual and situational symptom focusing, operationalized as attention towards neck muscle tension. The main experimental manipulation had no effect on indices of skin conductance, and data from the different experimental groups were combined for the present analysis, where we focus on the effect of the public speaking treatment. There were two baseline measurements, one measurement after the announcement of a public speech, and another after disclosure of the speech topic. This manipulation was carried out in order to separate effects of anxiety and cognitive load.

Dataset 2 included four measurements for each of 32 healthy female university students (19–29 years) who underwent a similar public speaking experiment in a between-subjects design. That is to say, half of the participants were to deliver a public speech, and the other half a speech without an audience. There was one baseline measurement, one measurement after announcement of the speech, and another after disclosure of the topic. Fourteen of 128 epochs contained motion artefacts and were excluded.

### Apparatus

After skin cleansing with propanol, skin conductance was recorded on thenar/hypothenar of the non-dominant hand using 8 mm Ag/AgCl cup electrodes (Coulbourn, Whitehall, PA) and 0.5% NaCl electrode gel (Par, Berlin, Germany) on thenar/hypothenar of the non-dominant hand; 0.5 V constant voltage was provided by a S77-21 coupler (Coulbourn). The signal was band pass filtered (0.015 and 5 Hz), digitally converted with 10 Hz (Dataset 1) or 100 Hz (Dataset 2) sampling rate (DI-205, Dataq, Akron, OH) and recorded (Windaq, Dataq). Each 60-s epoch was analyzed using a semi-automatic method (Event Detection and Analysis, Trosiener & Kayser, 1993) with a threshold of 0.25 μS. This analysis had already been performed in the context of the original experiments, before the present method was developed, and the corresponding results can be regarded as unbiased.

### Data Pre-Processing

Data analysis was carried out in Matlab (MathWorks, Natick, MA) using custom code that is available from the authors. After import of the 60-s segments into Matlab, the data were again low-pass filtered with a bidirectional first-order Butterworth filter at a cut-off frequency of 5 Hz, and re-sampled to 10 Hz (Dataset 2). No high-pass filtering was applied at this stage (note that data were high-pass filtered during recording).

### Statistical Analysis

The correspondence between conventional and DCM data analyses were summarized with Pearson correlation coefficients between the numbers of detected responses from both methods. Predictive validity was assessed as the correlation between the (known) psychological state and the estimated sympathetic arousal based on the number of responses. This number is estimated by thresholding the continuous estimates of SNA (DCM) or SF (conventional analysis).

For the training Dataset 1, the psychological state was defined for each epoch as either baseline or anticipation, and the estimated arousal as number of SNA responses for each epoch. For Dataset 2, which employed a between-subject design (anticipation of public versus anticipation of a non-public speech), psychological state was defined as public or non-public speech with arousal estimated by the mean number of responses in anticipatory epochs minus the number of responses in the baseline epoch. In one participant, the baseline epoch had been excluded such that *n*=31 for this analysis.

Relative sensitivity and specificity of the conventional and DCM analyses were quantified using receiver operator characteristics (ROC) curves. Predicting a discrete psychological state from a continuous variable can be reframed by drawing on signal detection theory (Macmillan & Creelman, 2005). Here we tried to classify a given state based on the total number of SF from the conventional analysis and the number of bursts estimated with DCM (both at an amplitude threshold of 0.1 μS). This allowed us to predict the true psychological state (and calculate specificity and sensitivity of that prediction) given the estimated number of responses. Finally, to test whether DCM estimates of autonomic arousal explain more variance in the psychological state than conventional estimates, we computed an *F*-statistic and its associated *p*-value by comparing two simple regression models for the two predictor variables. This *F*-statistic represents the amount of variance in psychological states that is explained by DCM above and beyond the conventional estimates.

## Results

As described previously, we analyzed the training dataset using an uninformed simple finite impulse response model to estimate a canonical response function (CRF). This requires knowing the onsets of the underlying bursts, which we approximated using a conventional semi-automated analysis of the SF time series (Bach, Friston, & Dolan, 2010). The ensuing CRF was used to optimize the parameters of the DCM so that its implicit convolution kernel matched the CRF (see Appendix). The resulting DCM was then used to deconvolve the time series. Figure 1C and D show DCM inversion for an exemplar epoch and give an overview of data fit and the estimated SNA generating the data. Figure 1E shows the correspondence between the estimated number of (above-threshold) SNA bursts and the SF number estimated by conventional analysis as a function of the threshold used to detect bursts. The correspondence between the two measures increases with increasing threshold and plateaus from a value of about 0.1 μS upwards.

**Figure 2:**
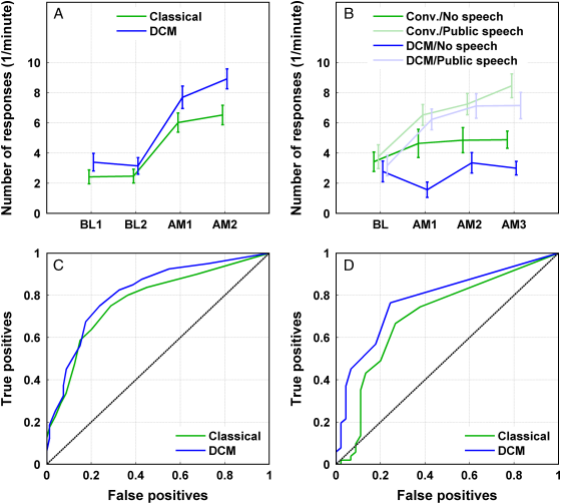
Number of responses estimated by conventional analysis and the DCM with a threshold of 0.1 μS. (A) Training dataset. BL: baseline measurements, AM1 after announcement of public speech, AM2 after announcement of speech topic. (B) Validation of the method on an independent dataset. BL: baseline measurement, AM1 and AM2 after announcement of speech, AM3 after announcement of speech topic. Solid line: non-public speech (control condition); dashed line: public speech. (C) ROC curves for the training dataset. (D) ROC curves for the second dataset.

In Figure 1F, we depict predictive (external) validity of both methods as the Pearson correlation between the number of estimated responses and the class (baseline or anticipation) to which the epoch belongs. The conventional analysis has better predictive validity at low thresholds. This probably reflects the fact that sustained responses (which could be due to peripheral factors alone) are modeled as sustained SNA. However, from around a threshold of 0.1 μS, validity of DCM response estimates is higher than that of the conventional method. For illustrative purposes, results from both methods for a threshold of 0.1 μS are shown separately for the four measurement periods in Figure 2A.

The ROC curves illustrating the trade-off between sensitivity and specificity at a threshold of 0.1 μS are shown in Figure 2C. An ROC curve that is closer to the upper left corner of the diagram indicates better prediction. Thus, the ROC curves point to higher validity of the DCM estimates.

We next validated the model using an independent dataset (Dataset 2) using the optimized parameters from Dataset 1. This is important since the CRF used to optimize the DCM parameters was derived from the same dataset to which the DCM was applied. Although the CRF was based on a large number of responses (1153 SF), its generalizability has to be confirmed.

Across the second dataset, the correlation between the number of responses (at a threshold of 0.1 μS) detected by conventional analysis and DCM was *r*=.67. Predictive validity (i.e., the ability to predict whether an individual was subjected to public speech anticipation or non-public speech anticipation) was *r*=.29 for the conventional and *r*=.50 for the DCM method. Thus, DCM estimates explained a higher proportion of variance than results from the conventional method (*F*(1,30)=6.6; *p*<.05). Results from both methods are depicted in Figure 2B for illustrative purposes, and Figure 2D corroborates the higher validity for DCM inversion in terms of ROC curves for this dataset.

## Discussion

In this paper, we present a dynamic causal model of skin conductance fluctuations SF and demonstrate that its inversion can be used to predict known psychological states. Crucially, our method showed a significantly higher predictive validity than that afforded by a conventional analysis. This advantage reflects the fact that sudomotor nerve firing is more closely related to the underlying psychological state than the ensuing SF, and suggests that SNA can be inferred from SF, using variational Bayesian inversion of our generative model.

We note a high correlation between both methods in the training dataset; an unsurprising observation given that the response function used to optimize the DCM was developed from a conventional analysis. This correspondence between the two methods was much lower in the second dataset, while at the same time the predictive validity of model inversion was relatively higher. Note that, in contradistinction to previously proposed approaches, our goal was not to emulate conventional analysis or perfectly fit the data, but to extract meaningful information about psychological states from the data.^1^ We were successful in this aim for both datasets, which necessarily led to a lower correlation with conventional methods.

Two factors may account for an enhanced predictive validity of our method: one is that any subjective element is removed from analysis, and the other is a suppression of noise through model constraints (i.e., parameterization of the unknown SNA). This contrasts with previous deconvolution approaches that try to recover unconstrained SNA estimates (Alexander et al., 2005; Benedek & Kaernbach, 2009;), an approach that might be more susceptible to measurement noise. An interesting extension of the model presented here would be to estimate the parameters of the DCM from the data being analyzed (as opposed to optimizing them using some estimated or assumed CRF, as in this paper). This might enhance the model fit, but possibly reduce the precision of the estimators of the neural states.

While inverting a DCM is computationally expensive, the ensuing quantification of the autonomic state is more precise than that afforded by previously proposed simple methods (i.e., area under the curve, Bach, Friston, & Dolan, 2010). Our DCM rests on physiological observations, which in part relate to biophysical models but are not entirely explained by such models. This means that the physiological realism of the DCM could be much improved. Nevertheless, our model can be generalized to any independent dataset acquired from healthy young populations with similar experimental set-ups. The generalizability to qualitatively different populations (e.g., patients) and measurement methods needs to be tested further. We have shown for event-related responses that one canonical response function can fit data from different recording sites (Bach, Flandin, Friston, & Dolan, 2010) and this might even be more tenable for SF, in which response latency is not an issue. On the other hand, since filtering influences the shape of the response, it seems crucial to use similar constant voltage measurement and filter settings as the ones applied here, in order to use our DCM parameters. In addition, when quantifying autonomic states from the DCM, the (arbitrary) amplitude threshold used here needs validation for different recording sites and measurement equipment. In general, we would like to encourage other researchers to refine the forward model. Different models can easily be compared in this framework by their likelihood, given the data, and by their predictive validity.

To our knowledge, this is the first report of a biophysically motivated generative model for peripheral physiological parameters of psychological states. Such dynamic causal modeling (Daunizeau, Friston, & Kiebel, 2009; Friston et al., 2003;) is becoming standard in neuroimaging, with applications for the analysis of fMRI, EEG/MEG (Chen, Kiebel, & Friston, 2008; Daunizeau, Kiebel, & Friston, 2009; David et al., 2006; Kiebel, Garrido, Moran, Chen, & Friston, 2009; Penny, Litvak, Fuentemilla, Duzel, & Friston, 2009;), and electrophysiological data (Moran et al., 2009). The power of such approaches lies in the estimation of causes and unknown (hidden) states by inversion of a mapping from causes to observations. This mapping enables one to place key biophysical constraints on the models and its associated estimators. Furthermore, the parameters and states of these models have a direct and useful biological interpretation. Thus, DCM allows for a wide range of possible implementation in psychophysiology, which we hope to exploit with this work.

## Footnotes

1 Actually, it is well known in the statistical community that overfitting, i.e., the tendency to fit both the signal and the noise in the data, leads to strongly biased estimation and inference (see, e.g., Carlin & Louis, 2000).

## APPENDIX

Our generative model comprises the following elements:

1. Each of *n* SNA bursts is modeled as a Gaussian function with a standard deviation of *σ*=0.3 s, while the amplitude *a* and the time of maximum firing *τ* are estimated from the data. The sum of these bursts is evaluated at each time point *t* and forms the parameterized input *u*(*t, θ*) to the skin conductance function:
  We assumed a fixed number of *n*=30 SNA bursts per minute. This is therefore the maximum number of detectable responses in the data. If there are fewer than *n* SN bursts in the data, the amplitude of any extra bursts would be estimated as zero.
2. The skin conductance time series is thought to result from a double convolution operation applied on the sudomotor nerve activity *u*(*t*, θ). This is modeled as a third-order ordinary differential equation (ODE) with parameters
  (1)
  where we have dropped explicit time notation, and *x* is related to the measured skin conductance time series *y* using the following (trivial) observation function:
  (2)

Here, *ɛ* is a residual error term. The parameters  were optimized so that they reproduced the canonical SF response function described previously (Bach, Friston, & Dolan, 2010), using the variational Bayes scheme described below: i.e., treating the canonical response function as data *y*(t)=*CRF*(*t*) and  as unknown parameters with input *u*(t)=*δ*(0). The ensuing posterior estimates of these parameters are:

Then, the model was inverted using a variational Bayesian inversion scheme described in Friston, Mattout, Trujillo-Barreto, Ashburner, and Penny (2007). In brief this entails:

- Using Gaussian assumptions about the residual errors in the observation process, Equations 1 and 2 are compiled to derive a likelihood function *p*(*y*|*θ*), which measures the likelihood of a set of observed SF *y*, given parameters *θ*.
- Defining *priors p*(*θ*) on the model parameters, which enable one to derive the *posterior* probability density function (pdf) over the evolution parameters:
  (3)

The posterior pdf *p*(*θ*|*y*) measures how likely any particular value of the unknown parameter *θ* is, given the measured times-series of SF.

- Having estimated the unknown parameters of the model, we can then define an estimator *û* of the unknown time-series of sudomotor nerve activity:
  (4)
